# Immobilization of M3 Muscarinic Receptor to Rapidly Analyze Drug—Protein Interactions and Bioactive Components in a Natural Plant

**DOI:** 10.3390/ijms24087171

**Published:** 2023-04-12

**Authors:** Hushuai Fan, Xiaomin Huang, Ziru Zhang, Ting Wang, Ludan Wang, Yajun Zhang

**Affiliations:** Key Laboratory of Resource Biology and Biotechnology in Western China, Ministry of Education, College of Life Sciences, Northwest University, Xi’an 710069, China

**Keywords:** immobilized receptor, M3 muscarinic receptors, drug–protein interaction, bioactive components, *Daturae Flos*

## Abstract

Despite its increasing application in pursing potential ligands, the capacity of receptor affinity chromatography is greatly challenged as most current research studies lack a comprehensive characterization of the ligand–receptor interaction, particularly when simultaneously determining their binding thermodynamics and kinetics. This work developed an immobilized M3 muscarinic receptor (M3R) affinity column by fixing M3R on amino polystyrene microspheres via the interaction of a 6-chlorohexanoic acid linker with haloalkane dehalogenase. The efficiency of the immobilized M3R was tested by characterizing the binding thermodynamics and kinetics of three known drugs to immobilized M3R using a frontal analysis and the peak profiling method, as well as by analyzing the bioactive compounds in *Daturae Flos* (DF) extract. The data showed that the immobilized M3R demonstrated good specificity, stability, and competence for analyzing drug–protein interactions. The association constants of (−)-scopolamine hydrochloride, atropine sulfate, and pilocarpine to M3R were determined to be (2.39 ± 0.03) × 10^4^, (3.71 ± 0.03) × 10^4^, and (2.73 ± 0.04) × 10^4^ M^−1^, respectively, with dissociation rate constants of 27.47 ± 0.65, 14.28 ± 0.17, and 10.70 ± 0.35 min^−1^, respectively. Hyoscyamine and scopolamine were verified as the bioactive compounds that bind to M3R in the DF extract. Our results suggest that the immobilized M3R method was capable of determining drug–protein binding parameters and probing specific ligands in a natural plant, thus enhancing the effectiveness of receptor affinity chromatography in diverse stages of drug discovery.

## 1. Introduction

A receptor is a functional protein that mediates signal transduction in cells. After a drug enters the body, it forms a stable complex with a receptor through affinity and then initiates the downstream signal transduction pathway to exert its efficacy. The binding of a ligand to a receptor is a molecular recognition process that relies on van der Waals forces, hydrogen bonds, and/or ionic bonds. The recognition behavior of a receptor to a drug is important for elucidating the mechanism of the action of drugs, discovering new drug targets, guiding clinical drug use, and developing innovative drugs [1,2].

Based on the theory of receptors, the study of a drug and its target receptors in vitro plays a key role in the assessment of pharmacodynamics. The measurement of drug–receptor associations can reveal the relevant coupling principle, afford a valuable theory for drug development [3]. Similarly, the binding interactions provide avenues for assays of bioactive compounds from complex matrices because of the specificity of a receptor to its ligands [4,5]. Thus, technologies that enable the rapid detection of drug–receptor associations and bioactive components in a complex mixture have caught the attention of chemists, biologists, and pharmacists.

Several modern techniques have been used to examine drug–protein interactions, including surface plasmon resonance [6], graph neural networks [7], multispectroscopic analysis [8], molecular docking and molecular dynamics simulations [9,10], and chromatographic methods [11]. Among these methodologies, quantitative affinity chromatography is extremely powerful. This method combines chromatographic separation and the specific binding capacity of the receptor protein [11,12], the advantages of which not only provide good specificity but also address the principle of drug–receptor interactions efficiently [13].

Despite the considerable advancement of G-protein-coupled receptor ligand research for drug discovery, the development of a muscarinic acetylcholine receptor (mAChR) binding system remains a challenge. There are five subisoforms of mAChRs (M1–M5) that cause differential physiological reactions [14,15]. Among them, the M3 muscarinic receptor (M3R) has been found to be expressed in the smooth muscle, bronchus, brain, and glands [16,17]. Such a widespread distribution makes this receptor a wonderful therapeutic target against multiple disorders, such as stomachache, asthma, and ulcerative colitis [18]. Thus far, the ligands of M3R have mostly been developed as synthetic orthostatic antagonists, such as scopolamine, atropine, and tiotropium [19]. They can bind to the corresponding receptor via sites of the endogenous neurotransmitter acetylcholine, consequently prohibiting the subsequent signaling of acetylcholine that causes certain disorders [20]. Although these drugs are widely employed in practice, the development of selective M3R antagonists is still limited due to the lack of a highly selective way to design or assay the ligands for the receptor.

In this study, we constructed a haloalkane dehalogenase (Halo)-tagged M3R in *Escherichia coli*. The immobilized Halo-tagged M3R was achieved by mixing cellular lysates and 6-chlorocaproic-acid-modified amino microspheres. The efficiency of the stable-phase labeling with the immobilized Halo-tagged M3R was examined via a receptor–drug association analysis by detecting the binding parameters of three known drugs to the receptor. Additionally, the active ingredients that couple to M3R in *Daturae Flos* (DF) with high specificity were identified. The results indicated that the immobilized M3R can be used to estimate drug–receptor associations and to determine active ingredients in a natural plant, demonstrating high repeatability, good stability, and precision.

## 2. Results and Discussion

### 2.1. Expression of Halo-Tagged M3R

**Figure 1:**
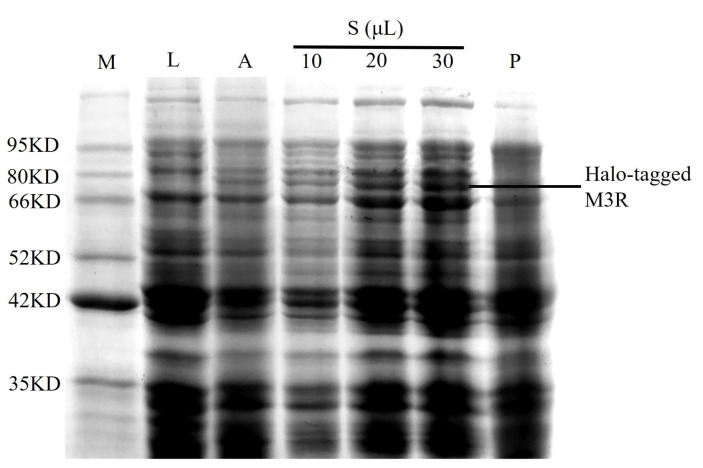
**Identification of M3R in *E. coli* expressing M3R.** Protein marker (M), 10 μL of Luria-Bertani medium (L), 10 μL of autoinduction medium (A), 10 μL, 20 μL, and 30 μL of cell lysate supernatant (S), and 10 μL of cell lysate precipitate (P) were loaded onto a 10% sodium dodecyl sulfate–polyacrylamide gel and stained with Coomassie blue. The corresponding molecular weights of the protein marker are indicated on the left side of the gel.

The M3R expression in *E. coli* lysates was analyzed using sodium dodecyl sulfate–polyacrylamide gel electrophoresis. A sharp band (73.4 kDa) was observed between 66.0 and 80.0 kDa in the autoinduction media sample; however, it was not found in the negative control lysate (Figure 1). Based on the theoretical calculations of M3R (40.4 kDa) and the Halo-tag (33.0 kDa), we considered that this band was the expressed Halo-tagged M3R. in addition to the increase of loaded sample from 10 μL to 30 μL, the band gradually became stronger. Such bands appeared mainly in the supernatant rather than the precipitate, implying that the expressed receptor is soluble because it mainly appeared in the aqueous supernatant but not in the sedimentary fraction.

### 2.2. Specificity and Stability Assessments of the M3R-Immobilized Column

**Figure 2:**
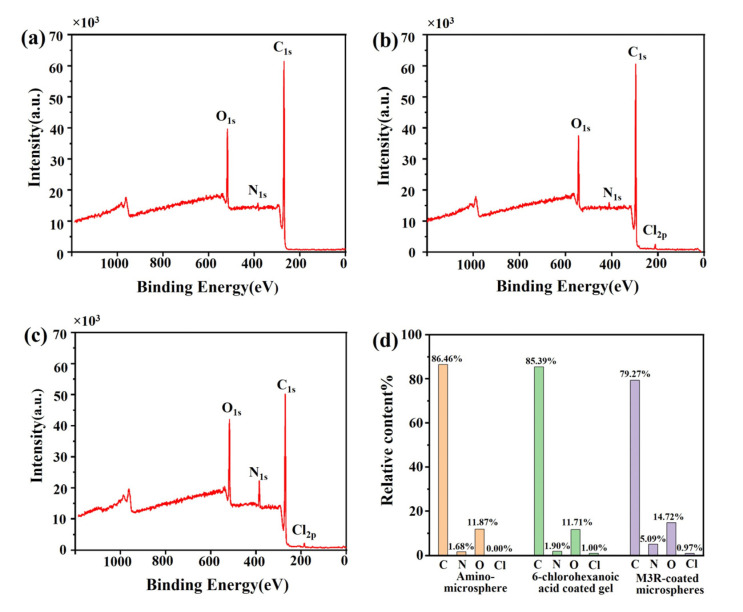
**The element characterization of amino microspheres by X-ray photoelectron spectroscopy.** The images show the element characterization of amino microspheres (**a**), 6-chlorohexanoic-acid-coated microspheres (**b**), and M3R-coated microspheres (**c**) by X-ray photoelectron spectroscopy (**d**). The percentage distribution of the elements C, N, O, and Cl in the amino microspheres, 6-chlorohexanoic-acid-coated amino microspheres, or M3-receptor-coated amino microspheres are calculated.

The amount of the immobilized M3R was analyzed by XPS to estimate whether the Halo-tagged M3R was successfully fixed on the microsphere surface. The peaks of the bare microspheres were at 517, 384, and 269 eV, respectively, which completely corresponded to O_1s_, N_1s_, and C_1s_ (Figure 2a). In the 6-chlorocaproic-acid-coupled microspheres, compared with the bare microspheres, the relative concentration of the C element was decreased. Due to the successful linkage to 6-chlorocaproic acid, the specific peak of Cl_2p_ occurred at 185 eV (Figure 2b). Due to the enhancement of amino and carboxylic acid groups in M3R, the relative amounts of N and O were tremendously elevated after the microspheres were bound to M3R (Figure 2c). Meanwhile, a further reduced amount of the C element indicated the successful binding of M3R as well. These results suggested that M3R was successfully bound to the microspheres.

**Figure 3:**
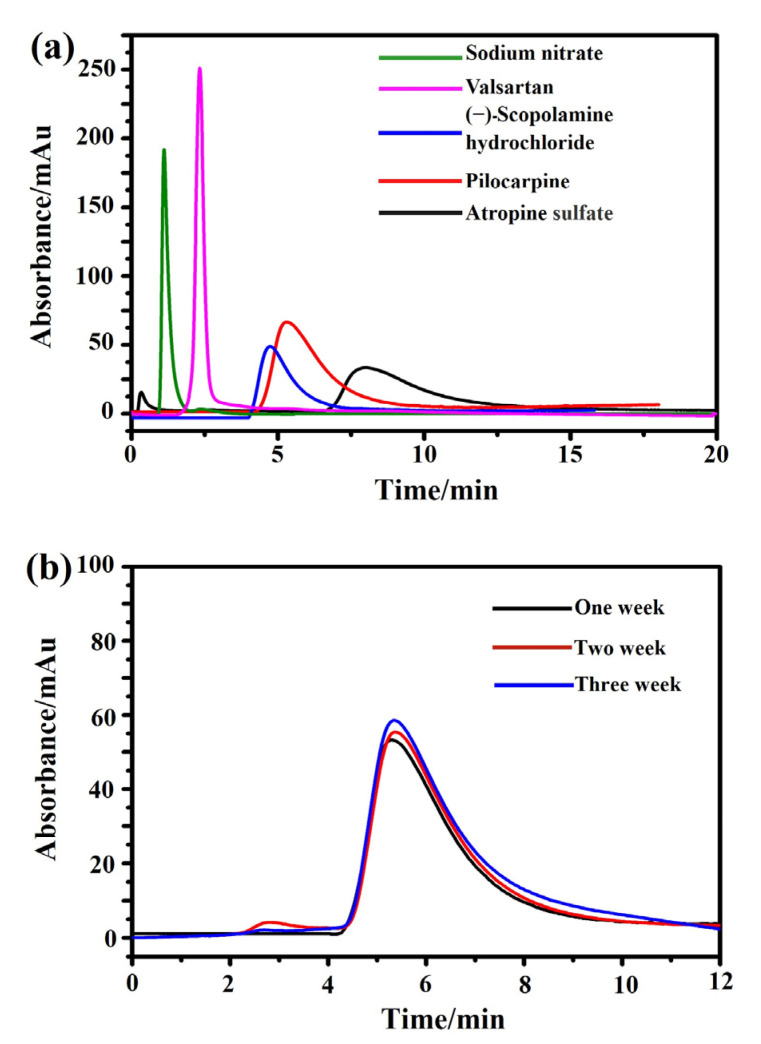
**Characterization of specificity and stability of the immobilized M3R by the ligand-binding assay.** (**a**) The chromatograms of atropine sulfate (brown), pilocarpine (orange), (−)-scopolamine hydrochloride (blue), valsartan (pink), and sodium nitrate (green) on the M3R-immobilized column. (**b**) The chromatograms of pilocarpine on the M3R-immobilized column in week one (brown), week two (orange), and week three (blue).

Next, the specificity and stability of the M3R-immobilized column were tested. The reference compound, sodium nitrite, had a retention time of 1.1 min on the M3R-immobilized column. However, as expected, the retention times of (−)-scopolamine hydrochloride, atropine sulfate, and pilocarpine were 4.7, 7.9, and 5.4 min, respectively, which were significantly longer than that of sodium nitrite, suggesting that receptor binding and specific association occurred among the drugs. Valsartan (an antagonist of type I angiotensin II receptor) had a retention time of 2.3 min, showing little difference from the system void time and indicating that no specific binding occurred on the column (Figure 3a). In summary, the retention times of both specific and nonspecific receptor ligands revealed that the M3R-immobilized column demonstrated specificity to bind the receptor’s ligands.

Next, the stability of the immobilized M3R was detected via the peak profiles and retention times of pilocarpine over three weeks. No significant alterations of the peak profiles and retention times were found, as indicated by their standard deviations of less than 5.0% (Figure 3b). Based on these results, we deduced that the immobilized M3R was stable for detecting the ligand retention times for 21 days, which is long enough to test drug–receptor interactions and bioactive compounds in herbs.

### 2.3. M3R-Immobilized Column for Determination of the Binding Coefficient of Drug–Receptor Interactions

#### 2.3.1. Association Constants and Binding Site Numbers

**Figure 4:**
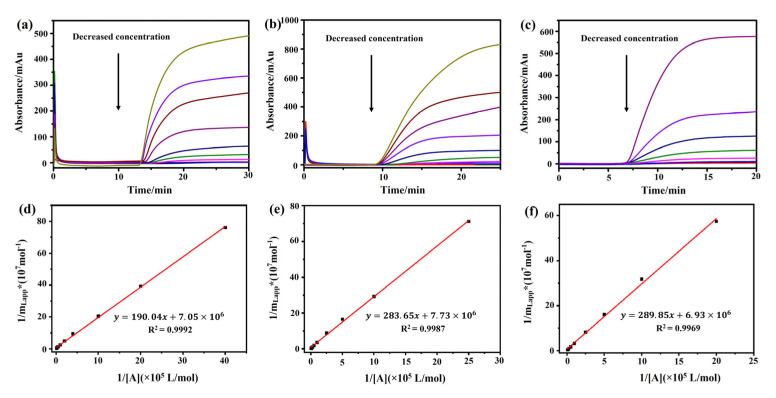
**Analysis of the association constants of atropine sulfate, pilocarpine, and (−)-scopolamine hydrochloride with M3R by frontal affinity chromatography**. The breakthrough curves of atropine sulfate (**a**), pilocarpine (**b**), and (−)-scopolamine hydrochloride (**c**) by the M3R-immobilized column. The regression curves of atropine sulfate (**d**), pilocarpine (**e**), and (−)-scopolamine hydrochloride (**f**) obtained by plotting 1/*m*_Lapp_ vs. 1/[*A*].

A frontal analysis was conducted with different concentrations of atropine sulfate, (−)-scopolamine hydrochloride, and pilocarpine. We observed that the breakthrough times of these three drugs decreased along with the increase in their concentrations (Figure 4a–c). A linear correlation was observed between 1/*m*_Lapp_ and 1/[*A*] of the drugs (Figure 4d–f, Table 1). The regression equations of (−)-scopolamine hydrochloride, atropine sulfate, and pilocarpine were *y* = 289.85*x* + 6.93 × 10^6^, *y* = 190.04*x* + 7.05 × 10^6^, and *y* = 283.65*x* + 7.73 × 10^6^, with correlation coefficients (*R*^2^) of 0.9992, 0.9969, and 0.9987, respectively. The binding parameters of the three drugs were estimated by Equation (1) and are summarized in Table 1. The frontal analysis showed that the binding constants of (−)-scopolamine hydrochloride, atropine sulfate, and pilocarpine to M3R were (2.39 ± 0.03) × 10^4^, (3.71 ± 0.03) × 10^4^, and (2.73 ± 0.04) × 10^4^ M^−1^, respectively. The high coefficients of the linear regressions suggested that there was only a single type of binding site for the drugs. The numbers of binding sites for (−)-scopolamine hydrochloride, atropine sulfate, and pilocarpine to M3R were calculated as (1.44 ± 0.19) × 10^−7^, (1.42 ± 0.45) × 10^−7^, and (1.29 ± 0.30) × 10^−7^ M, respectively. Therefore, the order of the M3R binding constants of these three drugs was as follows: atropine sulfate > pilocarpine > (−)-scopolamine hydrochloride.

#### 2.3.2. Dissociation Rate Constants

Peak profile analysis is a powerful method for determining the dynamics of drug–protein interactions. A previous study revealed that the concentration of the injected solute plays a crucial role in the apparent dissociation rate constant (*k*_d_) examined by peak profile analysis. The *k*_d_ value can be accurately detected once the flow rate is high enough to keep the term *κ* (kinetic factor) constant [21]. Therefore, an optimal flow rate is required to ensure the accurate measurement of kinetic parameters by a peak profile analysis. In this experiment, we chose flow rates of 0.4, 0.8, 1.2, 1.6, 2.0, 2.4, 2.8, 3.2, and 3.6 mL/min.

**Figure 5:**
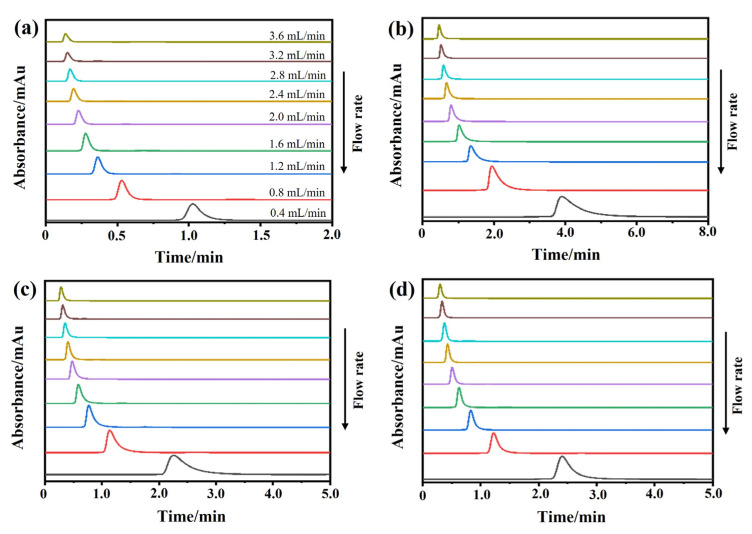
**Representative chromatograms.** The representative chromatograms of sodium nitrate (**a**), atropine sulfate (**b**), pilocarpine (**c**), and (−)-scopolamine hydrochloride (**d**) on the M3R-immobilized column at indicated flow rates.

**Figure 6:**
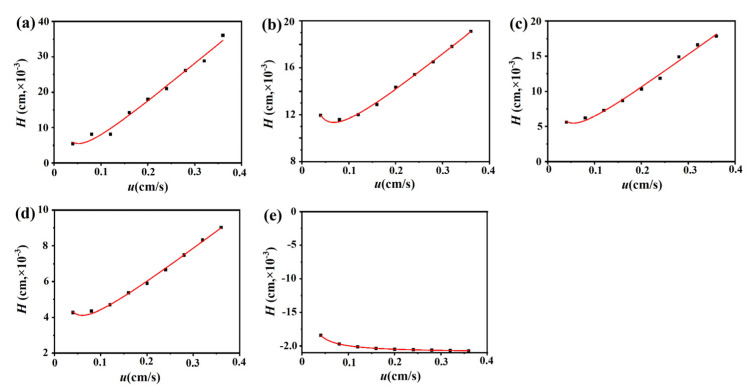
**Fitting plots of the three M3R-bound drugs, sodium nitrate, and the theoretical non-retained substance.** Fitting curves of the M3R-bound linear velocity (u) vs. plate height (H) of sodium nitrate (**a**), atropine sulfate (**b**), pilocarpine (**c**), (−)-scopolamine hydrochloride (**d**), and the theoretical non-retained substance (**e**).

**Figure 7:**
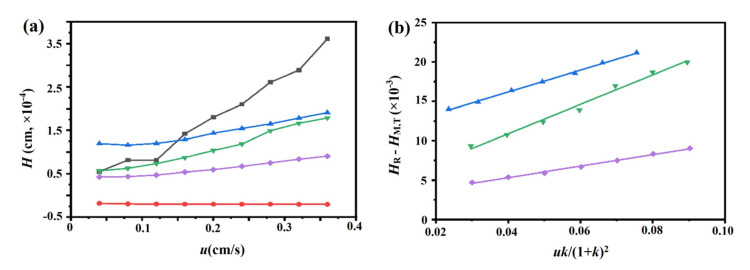
**Typical peak profiles of the three drugs, sodium nitrate, and the theoretical non-retained substance on the M3R-immobilized column**. The typical peak profiles of the three drugs, sodium nitrate, and the theoretical non-retained substance on the M3R-immobilized column by a multianalyte approach (**a**) and fitting plots of (*H*_R_−*H*_M_) vs. (*uk*)/(1 + *k*)^2^ for the binding of the three drugs to M3R (**b**). The blue triangle (▲) indicates atropine sulfate, the purple diamond (♦) represents pilocarpine, the green triangle (▼) is (−)-scopolamine hydrochloride, the black square (■) refers to sodium nitrate, and the red circle (●) represents the theoretical unretained substance.

The chromatograms of these three drugs at multiple flow rates are presented in Figure 5a–d. The retention times of (−)-scopolamine hydrochloride, atropine sulfate, and sodium nitrite were reduced as the flow rates were increased. The plate height plots of sodium nitrate and the three drugs on the M3R-immobilized column with multiple flow rates are presented in Figure 6a–d. The linear velocities were 0.04, 0.12, 0.16, 0.20, 0.24, 0.28, 0.32, and 0.36 cm/s, corresponding to flow velocities of 0.4, 0.8, 1.2, 1.6, 2.0, 2.4, 2.8, 3.2, and 3.6 mL/min, respectively. We observed that once the flow rate elevation was between 0.8 and 3.6 mL/min (0.08–0.36 cm/s), the *H*_R_ values of the drugs were obviously enhanced. The linear tendency of *H*_R_ vs. *u* suggests that a flow rate of 0.8 mL/min may be the minimal receivable flow velocity to determine the *k*_d_ value of drugs on the M3R-immobilized column. We also found that sodium nitrate (*H*_M_), as a non-retentive analyte, had a significantly greater plate height than the other three drugs. This could be caused by the widespread and slight nonspecific binding to the receptor and the obvious diffusion of sodium nitrate [22,23]. Therefore, a correction was necessary to obtain the actual plate height for the notionally non-retained substance (*H*_M,T_). After correction using Equation (3), it was found that the *H*_M,T_ value almost remained constant at a linear velocity range of 0.08–0.36 cm/s (Figure 6e). After merging the theoretically non-retained substance and the plate height curves of the three drugs, it was more clear that *H*_R_ was greater than *H*_M,T_ as the linear velocity was elevated to higher values (Figure 7a). The tendency between *H*_M,T_ and *H*_R_ was determined as anticipated by Equation (2). As a result, the typical plots of (*H*_R_–*H*_M,T_) vs. (*uk*/(1 + *k*)^2^) of the three drugs on the M3R-immobilized column at multiple flow rates are shown in Figure 7b. The linear regression equations for atropine sulfate, pilocarpine, and (−)-scopolamine hydrochloride were as follows: *y* = 140.0 × 10^−3^*x* + 106.8 × 10^−4^, *y* = 72.8 × 10^−3^*x* + 24.0 × 10^−4^, and *y* = 187.0 × 10^−3^*x* + 33.4 × 10^−4^, respectively. The correlation parameters (*R*^2^) spanned from 0.9867 to 0.9983. The dissociation rate constants of the immobilized M3R and the three drugs were estimated based on the slopes of the linear curves (summarized in Table 1). Their order was as follows: pilocarpine > atropine sulfate > (−)-scopolamine hydrochloride. Though greater than the data detected by radioligand binding, the *k*_d_ values revealed a similar ranking order [24] (Table 1).

Its ability to be applied at comparably high flow velocities is an asset of the peak profiling approach, which has become an optimal method for drug–protein association analysis in high-throughput screening. It can be applied in weak-to-moderate binding conditions (i.e., *K*_A_ ≤ 10^6^ M^−1^) [25]. The above results demonstrated that the association constants of the three drugs to M3R, calculated through frontal analysis, were less than 10^5^ M^−1^ (Table 1). The peak profiling approach was suitable for detecting the dissociation of the drugs from M3R in our study. Using the M3R affinity chromatography assay, we assessed the integration characteristics and mechanism between the receptor and the drugs, using frontal analysis and the peak profiling method as a mathematical model.

### 2.4. Identification of Bioactive Compounds in the DF Extract

**Figure 8:**
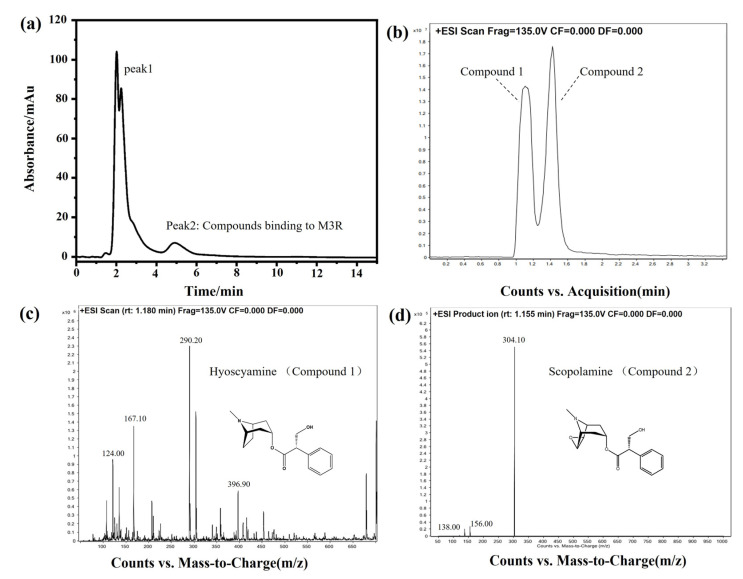
**Identification of bioactive compounds of *Daturae Flos* extract using the M3R-immobilized column.** (**a**) Representative chromatogram of the *Daturae Flos* extract on the M3R-immobilized column. Peak 2 was attributed to the compounds in the extract that specifically bind to M3R since the retention time was longer than the void time. (**b**) The total ion current of peak 2 in the chromatogram on the reversed-phase XDB-C_18_ column (2.1 mm × 150 mm, 3.5 μm) coupled with an electrospray-MS/MS system. (**c**) Compound 1 was identified as hyoscyamine by HPLC-MS/MS. (**d**) Compound 2 was identified as scopolamine by HPLC-MS/MS.

DF is the dried flower of *Datura metel* L. As an herbal medicine, DF has been applied to treat asthma, relieve pain, and smooth muscle spasms in traditional Chinese medicine clinical practice. Based on the pharmacological mechanism, we hypothesized that some bioactive components of DF can bind to M3R. Next, DF extracts were analyzed by the M3R-immobilized chromatographic column. Indeed, certain compounds did bind to the M3R-immobilized column. Two intense peaks of the chromatogram were observed (Figure 8a). Concerning two of the peaks, peak one was deemed a general mixture without any specific affinity to the immobilized M3R because the retention time was close to the void time of the column. Peak two had a longer retention time than the void time, indicating that the mixture coupled to M3R was coeluted. The following HPLC-Q-TOF-MS assay suggested that the eluted mixture was composed of two components, with the highest intensities at 290.20 ([M+H]^+^) and 304.10 ([M+H]^+^) corresponding to molecular weights of 289.20 and 303.10, respectively (Figure 8b–d). The daughter ions of these two ions (*m*/*z*) were 167.10 ([M–C_8_H_14_N]^+^) and 124.00 ([M–C_9_H_8_O_3_]^+^) as well as 156.00 ([M–C_9_H_8_O_2_]^+^) and 138.00 ([M–C_9_H_8_O_2_-OH]^+^). These compounds were verified as hyoscyamine and scopolamine [26,27].

A previous report indicated that the main active components in DF are tropane alkaloids, such as scopolamine, hyoscyamine, anisodine, anisodamine, et al. [28]. Our results showing that hyoscyamine and scopolamine were derived from DF were consistent with these findings. Hyoscyamine and scopolamine are also well-known agonists of mAChRs. These results demonstrate that the bioactive compounds of DF for treating asthma, relieving pain, and smoothing muscle spasms could be compounds such as hyoscyamine and scopolamine. The mechanisms underlying the anti-asthmatic and spasmolytic activity of hyoscyamine and scopolamine depend on the inhibition of M3R in effector cells as well as blocking the transmission of acetylcholine to relax the smooth muscle of the bronchus or other tissues. In summary, these results verified the accuracy of the method for analyzing the M3R-binding bioactive components in complex systems. Herein, we did not determine other compounds of the FD extract, including anisodamine and anisodine, using the M3R affinity column. Such results could be caused due to the low affinity of the subtype-selective ligands of M3R to the column or too low amounts of these compounds in the sample. The potential communication between M3R and other compounds requires further study.

## 3. Materials and Methods

### 3.1. Chemicals and Instruments

Reference standards of atropine sulfate (CAS: 55-48-1) and L-hyoscyamine sulfate (CAS: 620-61-1) were obtained from Xushuo Biotechnology (Shanghai, China). Valsartan (CAS: 137862-53-4) was purchased from Macklin Biochemical (Shanghai, China). Pilocarpine (CAS: 54-71-7) was purchased from MedChemExpress. (−)-Scopolamine hydrochloride (CAS: 55-16-3) was obtained from Aladdin (Shanghai, China). Ampicillin was obtained from Shanghai regal Biology Technology Co., Ltd. (Shanghai, China). Normal macroporous silica gel with a pore size of 300 Å and a particle size of 7.0 μm was obtained from the Shandong Bona Biological Technology Group (Jinan, China). The protein marker was obtained from Bitai Biotechnology (Shanghai, China). Sodium nitrite was obtained from Tianjin Kemio Chemical Reagent Co. Ltd. (Tianjin, China). DF was provided by the Wenzexuan Chinese Herbal Medicine Company. The other chemicals used were analytically pure, unless otherwise specified.

The VG Scientific ESCALAB220i-XL analyzer used for X-ray photoelectron spectroscopy (XPS) detection was from Thermo Scientific (Surrey, UK). The ZZXT-A wrapping machine was obtained from Elite Analytical Instruments (Dalian, China). The chromatographic system consisted of an Agilent 1100 high-performance liquid chromatography (HPLC)–ion trap mass spectrometer and an Agilent 1260 HPLC (Santa Clara, CA, USA).

### 3.2. Preparation of the M3R-Immobilized Column

The Halo-tag (GenBank accession: ADN27525.1) was synthesized by Sangon Biotech (Shanghai, China). The Halo-tagged M3R was expressed in *E. coli* BL21 (DE3), according to a previously described method [29]. Briefly, a single positive colony in an ampicillin (100.0 μg/mL)-containing semi-solid medium was picked up and incubated in 50 mL of ampicillin-containing Luria-Bertani medium at 37 °C. Fifteen hours later, the culture was transferred into auto-induction medium and incubated for another 12 h. After centrifugation, the collected cells were suspended in phosphate buffer, disrupted with an ultrasonic cell disruptor, and centrifuged to obtain the supernatant for the immobilization.

The immobilized M3R column was prepared through a bio-orthogonal reaction between haloalkane dehalogenase and chloroalkanes, which was described in a previous report [30]. Briefly, amino microspheres were reacted with γ-(aminopropyl) triethoxysilane and silica gel. The Schiff base method was employed to estimate the amino concentration on the resultant silica gel, and it showed that the concentration was 135.06 μmol/g. Then, 0.6 g of amino microspheres (1.0 equiv.) was soaked in 8.0 mL of 6-chlorocaproic acid (1.2 equiv., 14.94 mg) in *N,N*-dimethylformamide and reacted with 38.12 mg of 2-(7-azabenzotriazol-1-yl)-*N,N,N′,N′*-tetramethyluronium hexafluorophosphate (1.2 equiv.) and 42.77 μL of *N,N*-diisopropylethylamine (3.0 equiv.) for 4.0 h under stirring at room temperature. Next, the amino microspheres were collected and washed with methanol and phosphate buffer (20 mM, pH 7.4), successively. Finally, the prepared amino microspheres were mixed in 60 mL of a M3R-containing cell lysate for 1 h at 4 °C, filtered, rinsed with phosphate buffer (20 mM, pH 7.4), packed in a stainless steel column (4.6 mm × 30 mm) at a pressure of 300 bar, and stored in a refrigerator at 4 °C until their next use.

### 3.3. Characterization of the M3R-Immobilized Column

The elements of the microsphere surfaces were examined by XPS. As a stable phase control, both microporous silica gel and 6-chlorohexanoic-acid-coated microspheres were applied to eliminate the impact of the linker on the surface elements. Such alterations were used as probes to test whether the receptor was fixed on the microspheres.

A 5–10 nm layer of gold was covered on three types of dried microspheres under a vacuum. The elements O, C, and N on the surfaces of the microspheres were detected by a monochromatic Al Kα (1486.6 eV, 14.5 kV, 30 mA) in survey scanning mode. The binding energies were adjusted to the C_1s_ peak at 284.8 eV. The atom number per unit on the microsphere surface was set as the photoelectron value. Advantage software (Thermo Scientific) was applied for the XPS analysis. The corresponding atomic sensitivity factor and the characteristic peak area were employed to calculate the relative content of the target element.

### 3.4. Stability and Specificity Analyses of the M3R-Immobilized Column

The specificity of the M3R-immobilized column was analyzed by detecting the retention times of atropine sulfate, (−)-scopolamine hydrochloride (a muscarinic subtype nonspecific antagonist), pilocarpine (a specific agonist of M3R), and valsartan (an antagonist of type I angiotensin II receptor). Sodium nitrite, which does not specifically bind to protein, was employed to detect the void time of the chromatographic system. A relative standard deviation of >5.0% for the retention times between drugs was stipulated as an acceptable specificity. The detection wavelengths of sodium nitrite, atropine sulfate/pilocarpine, and (−)-scopolamine hydrochloride were set at 254 nm, 217 nm, and 210 nm, respectively. Phosphate buffer (20 mM, pH 7.4) was the mobile phase, and was kept at a flow rate of 0.2 mL/min. The injection volume was 10 μL. The stability of the immobilized M3R was assessed by analyzing the retention times and the peak profiles of (−)-scopolamine hydrochloride for three weeks.

### 3.5. Receptor–Drug Interaction Analysis

#### 3.5.1. Frontal Analysis

Similar to previous reports, the numbers of the binding sites of the isolated M3R-bound compounds and the affinity constants were detected by frontal analysis [31,32]. This detection was based on the mean shift of the breakthrough curves to the shortest breakthrough times during infusion of the M3R-immobilized column with enhanced concentrations of compounds. Different concentrations of three ligands were supplied into the mobile phases, and the M3R-immobilized column was continuously rinsed until stable absorptions were obtained. The concentrations of atropine sulfate used were 0.25, 0.5, 1.0, 2.5, 5.0, 10.0, 20.0, 40.0, 50.0, and 80.0 μM; those of pilocarpine were 0.4, 1.0, 2.0, 4.0, 10.0, 20.0, 40.0, 100.0, and 160.0 μM; and those of (−)-scopolamine hydrochloride were 0.5, 1.0, 2.0, 4.0, 10.0, 20.0, 40.0, and 100.0 μM. Each ligand concentration was assayed in triplicate. The corresponding breakthrough curves were documented. The binding parameters were estimated via Equation (1) [33]:

(1) $$\frac{1}{m_{Lapp}}=\frac{1}{m_{L}\times K_{A}}\times \frac{1}{\left[A\right]}+\frac{1}{m_{L}}$$

where *m*_Lapp_ is the molar concentration of the solute at the inflection point of the breakthrough curve, *K*_A_ is the drug’s affinity constant to the protein, [*A*] is the molar concentration of the detection compound in the mobile phase, and *m*_L_ is the number of binding sites. Based on the Equation (1), the curve of 1/*m*_Lapp_ vs. 1/[*A*] will have a slope of 1/(*m*_L_*K*_A_) and a y-intercept of 1/(*m*_L_). The affinity constant can be estimated by the ratio of the intercept to the slope. The number of binding sites was calculated by the inverse of the y-intercept.

#### 3.5.2. Peak Profiling Method

We used the peak profiling method to probe the binding kinetics of the drug–receptor interaction. The dissociation rate constant of each drug from the immobilized M3R was tested with this approach. Here, the applied concentrations of atropine sulfate, (−)-scopolamine hydrochloride, pilocarpine, and sodium nitrate were 0.3, 0.3, 0.5, and 0.1 mM, respectively. We used multiple flow rates of 0.4, 0.8, 1.2, 1.6, 2.0, 2.4, 2.8, 3.2, and 3.6 mL/min but avoided an extremely high flow rate.

Through peak profiling with multiple flow rates, the apparent dissociation rate constants of the drug–protein interactions were determined by Equation (2) [33,34]:

(2) $$H_{R}-H_{M}=\frac{2uk}{k_{d}{\left(1+k\right)}^{2}}$$

where *H*_R_ and *H*_M_ represent the solution’s total plate height as well as the non-retained substance on the column, respectively, *u* refers to the linear velocity of the mobile phase, and *k* indicates the analytic retention factor. The *k*_d_ value can be estimated through plotting the curve of (*H*_R_−*H*_M_) vs. (*uk*)/(1 + *k*)^2^, which leads to an inverse linear relationship of the slope and *k*_d_.

An amendment of *H*_M_ (*H*_M,T_) was applied to calculate the plate heights for inferential non-retained substances on the M3R-immobilized column by Equation (3) if *H*_M_ could not be detected directly by the conventional non-retained substances [22,23,35]:

(3) $$H_{M,T}=A_{C}+\frac{B_{C}}{\lambda \cdot u}+C_{sm,T}\cdot u$$

where λ represents the correction coefficient, which can be determined from the B-term proportion for the inferential non-retained substance and that of the usual non-retained substance, *B*_C_ and *A*_C_ are the B-term and A-term for the usual non-retained substance, respectively, and *C*_sm,T_ represents the stagnant mobile phase mass transfer involving the C-term for the inferential non-retained substance.

### 3.6. Analysis of the M3R-Binding Bioactive Components in the DF Extract

#### 3.6.1. Preparation of the DF Extract

**Figure 9:**
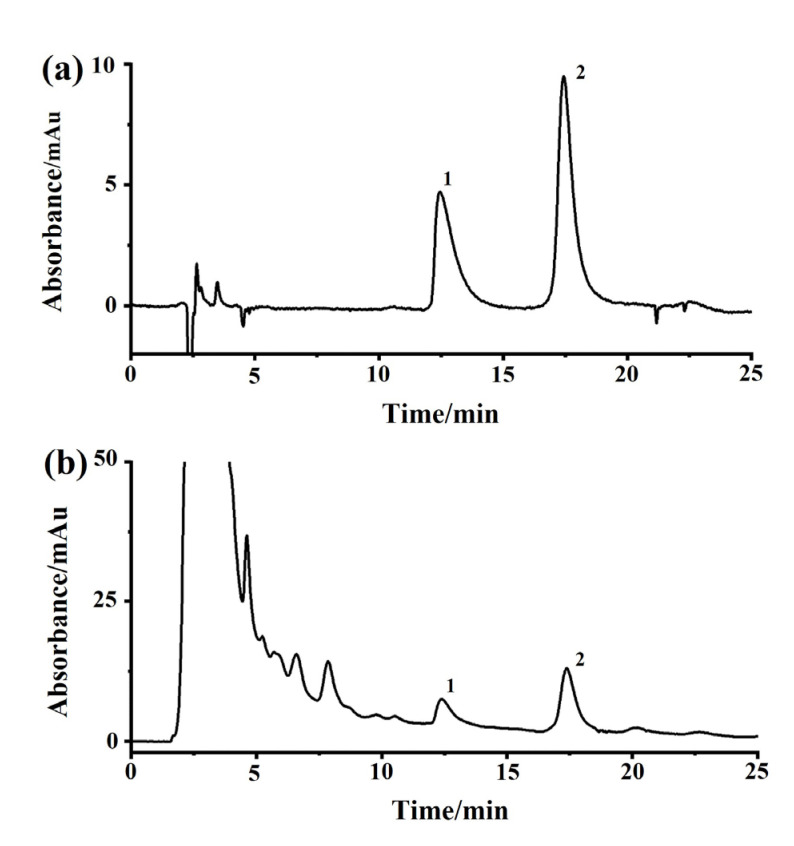
**Representative chromatograms of *Daturae Flos* extract on a reversed-phase column with a detection wavelength of 216 nm**. (**a**) Reference standards of atropine sulfate and (−)-scopolamine hydrochloride. (**b**) Extract of *Daturae Flos*; 1, atropine sulfate; 2, (−)-scopolamine hydrochloride.

The heating under reflux method was used to make the anhydrous extract of DM. In brief, 10 g of dried DF was soaked in 100 mL of water for 0.5 h, boiled for 2 × 1 h, filtered, and concentrated to 10 mL via rotary evaporation at 60 °C under reduced pressure. A solution of 95% ethanol was applied to carry out alcohol sedimentation to reduce the alcohol content of the concentrated extract to 75%. The liquid supernatant was concentrated at 60 °C to obtain an herbal solution of 1.0 g/mL. As shown in Figure 9, the concentrations of scopolamine and hyoscyamine in the extracted solution were condensed to 0.22% and 0.08% of the raw herb through the processes described in the Chinese Pharmacopoeia (Pharmacopoeia of PR China, 2020).

#### 3.6.2. Identification of Active Compounds in the DF Extract

The M3R-immobilized column was utilized to analyze the active components in the DF extract with an Agilent 1100 chromatographic system. The mobile phase was 5 mM ammonium acetate buffer, the flow rate was 0.3 mL/min, the sample volume was 10 μL, and the detection wavelength was 254 nm. The chromatographic peak, which had a retention time that was longer than the void time, was collected and considered the M3R-targeted bioactive compound. It was further analyzed using high performance liquid chromatography (HPLC)–mass spectrometry (MS).

An Eclipse XDB-C_18_ column (2.1 × 150 mm, 3.5 μm) was employed for chromatographic analysis with a 0.1% formic acid solution and methanol (40:60, *v*/*v*) as the mobile phase, a flow rate of 0.3 mL/min, and a column temperature of 30 °C. The MS analysis was carried out in both negative and positive modes with a scan range of 100–2000 amu, a nebulizing gas pressure of 30 psi, a flow rate of 8.0 L/min, and a dry gas temperature of 32.5 °C, respectively.

## 4. Conclusions

Taking M3R as a probe, this work illustrates how the immobilized receptor column could be used to analyze the binding thermodynamics and kinetics of drug–receptor interactions and to screen bioactive compounds from a natural plant that target the receptor. Due to its good specificity and stability, immobilized M3R was applied to determine the binding thermodynamics and kinetics of (−)-scopolamine hydrochloride, atropine sulfate, and pilocarpine to the receptor. Additionally, using the immobilized receptor, hyoscyamine and scopolamine were screened and verified as the bioactive compounds from the DF extract that bind to M3R. By using such a strategy, it is possible to enhance the effectiveness of receptor affinity chromatography in discovering and assessing a ligand. This offers receptor affinity chromatography the capacity to address the issues of current techniques, such as the non-specific adsorption of a drug/protein, the large volume of a sample, a pressure-mediated volume change, or a long incubation time. As such, we concluded that receptor immobilization is a favorable and promising approach for analyzing drug–protein interactions and probing bioactive components from complex matrices, including natural products.

## Figures and Tables

**Table 1:** The association constants (*K*_A_), numbers of binding sites (*m*_L_), and dissociation rate constants (*k*_d_) of atropine sulfate, pilocarpine, and (−)-scopolamine hydrochloride determined by M3R-immobilized column chromatography.

Ligands	*K*_A_ by Frontal Analysis (M^−1^)	*m*_L_ by Frontal Analysis (M)	*k*_d_ by the Peak Profiling Method (min^−1^)	*k*_d_ by the Radioligand Binding Assay (min^−1^)
Atropine sulfate	(3.71 ± 0.03) × 10^4^	(1.42 ± 0.45) × 10^−7^	14.28 ± 0.17	1.12 ± 0.13
Pilocarpine	(2.73 ± 0.04) × 10^4^	(1.29 ± 0.30) × 10^−7^	27.47 ± 0.65	15.3 ± 2.30
(−)-Scopolamine hydrochloride	(2.39 ± 0.03) × 10^4^	(1.44 ± 0.19) × 10^−7^	10.70 ± 0.35	0.31 ± 0.03

## Data Availability

All relevant data are within the paper.
